# ‘Where is the help for me here?’ Female domestic violence survivors’ narratives of navigating and receiving secondary-care mental health services in the United Kingdom

**DOI:** 10.1177/17455057251336142

**Published:** 2025-08-17

**Authors:** Megan Webb, Anne Cooke, Angela Sweeney

**Affiliations:** 1 Salomons Institute for Applied Psychology, Canterbury Christ Church University, UK; 2 Institute of Psychiatry, Psychology & Neuroscience, King’s College London, England

**Keywords:** domestic abuse, mental health, narrative, survivor, trauma-informed care

## Abstract

**Background:**

Mental health (MH) services are uniquely positioned not only to contribute to the identification of domestic violence but also to provide support. Survivors of domestic violence regularly access secondary-care MH services. Although several studies and reviews have been conducted globally, the limited literature exploring survivors’ experiences of MH services in England has focused on the acceptability of routine enquiry within MH services, MH service responses to domestic violence and abuse (DVA), and the facilitators and barriers to disclosure within community MH teams.

**Objectives:**

This study explores domestic violence survivors’ narratives of navigating and receiving secondary-care MH services in the United Kingdom.

**Design:**

A qualitative study was undertaken with seven women who had experienced domestic violence. The study adopted a feminist, qualitative, narrative approach to enable in-depth exploration of survivors’ accounts of secondary-care MH services.

**Method:**

Seven participants were recruited from three community-based third sector DVA services in the United Kingdom. Women participated in a face-to-face narrative inquiry interview ranging from 45 to 90 min. Interviews were transcribed verbatim and analysed in two distinct phases using the Voice-Centred Relational Method and thematic analysis, respectively.

**Results:**

Results suggest that MH professionals (MHPs) consistently missed the connection between abuse and women’s presenting distress. The structure of MH services resulted in superficial interventions and relationships, with key windows of opportunity for support missed. Whilst all women found a ‘safe haven’ where they felt heard, understood and supported, this was consistently outside of statutory MH services.

**Conclusion:**

Women’s experiences of secondary-care MH services often paralleled their experiences of abuse and were experienced as re-traumatising. Our findings suggest that adopting trauma-informed approaches, developing more integrated support, and considering alternative conceptualisations of distress beyond diagnosis could enable survivors to find safety and support.

## Introduction

Domestic violence and abuse (DVA) is a form of gender-based violence that disproportionally affects women and is recognised as a global public health issue.^1^ Globally, over a quarter of women aged 15–49 years who have been in a relationship have been subjected to physical and/or sexual violence by their intimate partner at least once in their lifetime, and as many as 38% of all female homicides are committed by intimate partners.^1^ In the United Kingdom, one in four women will experience DVA in their lifetime, and in England and Wales, two women a week are killed by current or ex-partners.^2^ Although anyone can experience DVA, women are more likely to experience repeated severe physical and sexual violence, coercive control, and domestic homicide.^3,4^ This study, therefore, focuses on female survivors defined as individuals who identify as women (cis- and transgender) and have experienced domestic violence. The terms ‘domestic abuse’ and ‘domestic violence’ are both used in the literature and in this article the acronym ‘DVA’ will be used. DVA can be defined as ‘an incident or pattern of incidents of controlling, coercive, threatening, degrading and violent behaviour, including sexual violence, in the majority of cases by a partner or ex-partner, but also by a family member or carer’.^4^ The abuse can encompass, but is not limited to: psychological, physical, sexual, financial, emotional abuse, and coercive control. Throughout this article, mental health professionals (MHPs) have been referred to as MHPs and mental health (MH) has been abbreviated to MH.

### Domestic violence and mental health

It is well-documented that DVA impacts psychological well-being and is associated with various presentations of distress, including anxiety, depression, post-traumatic stress, eating distress, psychosis, and substance use.^5–11^ Women who experience DVA are three times more likely than others to attempt to end their lives, and this is exacerbated for women who have experienced systemic inequalities such as poverty, racism, and ableism.^12^ Research has identified a bidirectional relationship between DVA and MH whereby experiencing DVA increases the likelihood of a later MH diagnosis, and individuals who receive an MH diagnosis are more likely than others to experience abuse.^7–9^ One potential explanation for this bidirectionality is that perpetrators can use women’s MH status as a coercive tool to threaten, undermine and denigrate their sense of self and credibility.^1,4^

### Experiences of mental health services

Within the United Kingdom, despite the link between MH and DVA, and mandates to ask about DVA (‘routine enquiry’), MH services have shown limited engagement in identifying or responding to DVA.^13,14^ Healthcare policies have sought to address this, such as through the Pathfinder project, the UK Government’s ‘Tackling Domestic Abuse Plan’, and the development of a National Institute for Care Excellence quality standard for responding to DVA.^15–17^ Yet female survivors who have accessed MH services consistently report being disbelieved and unheard.^18,19^ A review of experiences of disclosing DVA in MH settings found that MHPs did not consider the role of DVA in precipitating or exacerbating distress.^20^ This is consistent with reports that MH services are ‘not conducive to the disclosure of DVA’ (p.189) and that following a disclosure, experiences of DVA are rarely considered within care plans.^21^

Globally, several studies have investigated DVA survivors’ experiences of MH services across varying populations (e.g. veterans) and professional groups (e.g. psychologists, counsellors).^18,22–24^ In the United States, López-Aybar et al. found that DVA survivors faced discrimination and stigma from MHPs because of their DVA survivor status.^23^ For Black and minoritised participants, this was further compounded by experiences of racism and structural inequality in accessing MH services. Marsden et al. explored DVA survivors’ experiences of working with psychologists in Australia.^18^ Survivors reported that interactions with psychologists were experienced as harmful and re-traumatising as professionals often replicated abuse dynamics by imposing blame, disbelief, or their own assumptions. International studies have also reported a lack of communication and coordination between MH and DVA services consistent with concerns that there are significant gaps in service provision for DVA survivors experiencing long-lasting and high levels of distress.^4,18,19,22^

In the United Kingdom, two projects have explored DVA survivors’ experiences of secondary-care MH services. Within one qualitative project, Rose et al. explored the facilitators and barriers to disclosure from service user and professional perspectives.^21^ However, in an overlapping but slightly larger dataset, Trevillion et al. focused on attitudes towards routine enquiry for both service users and MHPs, and how service responses to DVA (e.g. identification of DVA, MHPs responses and referral pathways) could be improved within secondary-care MH services.^25^ Mantovani and Allen conducted a mixed-methods study to explore the prevalence of DVA within secondary-care MH services in a London borough.^26^ Focus groups with service users and stakeholders explored the facilitators and barriers to the identification of DVA, including the acceptability of routine enquiry, service responses to DVA, and attitudes towards the introduction of a peer-mentoring service. All studies found that routine enquiry was acceptable to participants and was facilitated by supportive and trusting relationships with MHPs. Some providers, however, reported hesitancy regarding routine enquiry due to lack of confidence, unclear referral pathways, and current pressures on MH services. The dominance of the medical model and diagnostic treatment was reported across studies as overshadowing the exploration of DVA and consistently acted as a barrier to disclosure. Survivors also reported not being believed or taken seriously by professionals because of assumptions about their MH diagnoses.

### Why are mental health services experienced as unhelpful?

It has been argued for decades that the current biomedical dominance in the Global North shapes people’s experiences of MH services, disconnecting socio-political and domestic contexts from survivors’ presenting distress.^27^ Biomedical approaches typically conceptualise ‘mental disorders’ as biologically based brain diseases, potentially triggered by environmental conditions, with pharmacological treatments seen to target presumed biological abnormalities/deficits.^28^ Much of the research in DVA focuses on the MH consequences of abuse and the likelihood of receiving various psychiatric diagnoses.^29,30^ The focus of interventions within MH services can, therefore, become about treating ‘the illness’ separate from the experience of DVA. This can result in inappropriate interventions that exacerbate women’s distress, compromise their safety, and fail to meet their needs.^31^

The language and principles of the biomedical model also have profound implications for how people make sense of their distress, life experiences, and overall identity.^32,33^ Survivor movements, trauma-informed theorists, and others have sought to reconnect people’s distress to their experiences and circumstances. They have argued that many ‘disorders’ are better recognised as understandable responses to trauma, and conceptualising these responses as ‘mental illnesses’ or ‘MH problems’ creates an individualistic focus that ignores wider social contexts and perpetrator actions.^34,35^

Trauma-informed care – a process of organisational change that seeks to prevent re-traumatisation and replication of abuse within services and create the conditions for healing – arose out of the recognition that many people accessing MH services will have experienced trauma and abuse and that services were not meeting the needs of survivors.^36^ Despite trauma-informed care being recommended within MH service policy in many countries, it has been argued that in practice, certainly in the United Kingdom, system-wide change has rarely been enacted.^37^

Several studies have explored what DVA survivors have found helpful when accessing MH care. In Australia, Marsden et al. explored survivors’ experiences of working with psychologists and found that trauma and violence-informed approaches were helpful, defined as believing survivors’, offering a safe space to talk, providing connection and centring the woman.^18^ Kastrani et al. found that positive therapeutic relationships with counsellors in Greece were characterised by safety, closeness and acknowledgement of DVA within its socio-political context.^24^ Sorrentino et al. explored DVA survivors’ experiences of MH services within a veteran population in the United States.^22^ They found that MH services were experienced as helpful when care was attuned to survivors’ needs, were flexible, and MHPs understood the complexity and nuance of DVA, whilst respecting survivors’ autonomy in decision-making. Similarly, in the United Kingdom, survivors reported acknowledgement of DVA, receptiveness to DVA disclosure, adoption of a non-discriminatory approach, and support for multiple needs as helpful factors in interactions with MHPs in a community mental health team (CMHT).^25^

### Rationale and aims

The National Health Service (NHS) provides the majority of MH services in the United Kingdom. Services are organised into primary, secondary and tertiary care, with secondary-care including community-based and inpatient specialist MH services. Secondary-care is typically provided to individuals experiencing long-lasting and high levels of distress, including many who receive a diagnosis of emotionally unstable personality disorder (EUPD), a diagnosis highly associated with experiences of abuse and disproportionately given to women.^38,39^ People accessing secondary-care MH services are two to eight times more likely than others to experience DVA and the lifetime prevalence of DVA in inpatient and outpatient MH services is 30%–60% and 33%, respectively.^7–9,29^

This study focuses on people using secondary-care MH services because there is a high prevalence of DVA in this population, and an increased risk of service responses replicating abuse dynamics, due to increased powers for restrictive practice (e.g. community treatment orders, detention under the Mental Health Act).^40^ Whilst people who had accessed primary and tertiary services were not excluded, interviewing people who have accessed secondary-care gives a perspective on this unique setting amongst people who are likely to experience long-lasting and high levels of distress.

Existing UK studies that have explored survivors’ experiences of MH services have largely focused on the acceptability of routine enquiry, the identification of DVA and MHPs responses, and experience of disclosure within CMHTs, rather than women’s narratives of their journeys through MH services (i.e. regardless of disclosure).^21,25,26^ To the best of our knowledge, studies that have explored experiences of longer-term MH support and experiences of navigating MH services have been conducted outside of a UK context.^18,24^ Given that MH services are structured, and may therefore be experienced differently internationally, and given concerns that there are significant gaps in service provision for DVA survivors experiencing long-lasting and high levels of distress in the United Kingdom, the main aim of this study was to explore DVA survivors’ narratives of navigating and receiving secondary-care MH services in England to understand survivors’ needs and inform service provision.^4,18,19^

## Method

### Positioning and design

The study adopted a feminist, qualitative, narrative approach to enable in-depth exploration of survivors’ accounts of secondary-care MH services.^41,42^ A key aim of feminist and narrative approaches is to reduce ‘power-over’ relationships between researchers and participants.^43,44^ Feminist research seeks to centre the lived experiences of women and girls, challenge dominant sources of knowledge, and place the lives of women and other marginalised communities at the centre of analyses. A further principle of feminist methodology is advancing social justice and change.^44^ Accordingly, the current research prioritised survivor voices to inform service provision. Given the topic, a trauma-informed approach to research was implemented to reduce the risk of harm.^45^ To support this, project design was informed by the ‘Survivors Voices Charter’ and the Women’s Aid ‘Research Integrity Framework’ which set out criteria for working with survivors safely.^46,47^ Standards for reporting qualitative research were followed.^48^

### Participants

Seven participants were recruited from three community-based third sector DVA services in Southern England. In the United Kingdom, DVA services are largely provided by third-sector organisations (i.e. charities, voluntary groups, social enterprises) that often rely on local authority funding to provide specialist outreach, refuge, advocacy and counselling to survivors. DVA services invited women to participate who met the study’s inclusion criteria. Recruiting women through third-sector DVA services meant women could access support pre- and post-interview if required. Participants were included in the study if they identified as women, were aged 18 and over, had experienced DVA, and had accessed secondary-care MH services. Exclusion criteria were (a) unable to provide informed consent and (b) unable to participate safely. This was decided in consultation with the participant and with the guidance of their DVA service. Conversations about what participation would entail enabled women to make informed decisions about the safety of participation and what was right for them.

### Procedure

Recruitment took place over 12 months between May 2022 and May 2023. Prospective participants were contacted using their specified safe contact method to discuss potential participation. After having time to consider participation, a safety plan and interview location were agreed. Measures were enacted to ensure interviews were trauma-informed including: offering interview questions in advance with a suggestion for participants to consider what they felt comfortable sharing, not asking about participants experiences of abuse at any point and reminding participants of processes regarding confidentiality and their right to withdraw. Interviews took place in safe and confidential spaces: for six participants, their preference was within the DVA service building, and for one participant, this was an online videoconferencing platform.

### Data collection

Women participated in a face-to-face narrative inquiry interview with the first author (MW) ranging from 45 to 90 min. Participants were asked to recall, in their own words, ‘what brought them into contact with secondary-care MH services for the first time’ and whether they felt their experiences of DVA had impacted their experiences of care. Participants were given space to speak freely. When narratives came to a natural pause, prompts such as ‘what happened next’ and ‘can you tell me more about that’ were used to support narrative flow.^49^ Participants were then asked specific open-ended questions about their journey through services. A debrief was provided at the end of each interview. Participants were given the opportunity to receive and review their transcripts and the analysis summary before project completion.

### Analysis

Interviews were audio-recorded, transcribed verbatim and analysed in two distinct phases.^50,51^ The Voice-Centred Relational Method (VCRM) of data analysis involved four readings of individual transcripts, following the guidelines of Hutton and Lystor: the first to understand what happened and consider the first author’s response; the second to focus on how women experienced, felt and spoke about themselves; the third for their relationships to others; and the fourth for their relationships to broader social, structural and cultural contexts.^52^ By listening for different ‘voices’ (through multiple readings of individual transcripts), VCRM aims to delay the reductionist stage of coding, helping to centre the ideas and experiences of participants rather than ‘confirming what the researcher knows’ (p.20).^53^ Differences across participants are maintained by considering their contexts and generating insights that may not emerge in a thematic approach alone.^54^

As in Montgomery et al. the study aimed to inform clinical practice, and as this was an explicit motivation for participation, a fifth reading was conducted using thematic analysis.^50,51,54^ Data were synthesised into a more readily accessible form, following the six-stage process of Braun and Clarke.^55^ Individual narratives were read in-depth and analysed line-by-line to develop initial codes using ‘in vivo’ and process coding, supported by Nvivo qualitative software. Narratives were compared to identify links, similarities and differences leading to the development of early themes. Member-checking validated the emerging findings through written and verbal feedback. All participants were offered the opportunity to review the emerging themes and subthemes. Three out of seven participants responded; all stated that the emerging themes and subthemes aligned with their experiences and therefore no further changes were required. Participants were also sent a summary of the study outlining the overall findings, which received positive feedback from four participants.

### Reflexivity and positionality

Reflexivity is a significant aspect of narrative research.^44,56^ VCRM acknowledges that however participant-led research may be, the researcher retains a significant role in initiating, facilitating and constructing meanings within the data. It is acknowledged that interview data is a co-construction between interviewer questions and what participants choose to share.^57^ Analysis was conducted by one author (MW), but several strategies were adopted in an ongoing reflective process to consider pre-existing beliefs and positioning relevant to the research, including a reflective diary, individual summaries, a bracketing interview with a narrative researcher and regular supervision.^58^ The first author (MW) undertook the study and carried out data collection as a cisgender, heterosexual, White woman who has experience of working within both the domestic violence sector and supporting survivors in secondary-care MH services. The study was undertaken as part of the first author’s training to be a clinical psychologist. The first author discussed and reflected with a supervisory team (AC and AS) with expertise in trauma-informed approaches. Readers are invited to consider this when drawing their conclusions.

### Ethics

Ethical approval was obtained from Canterbury Christ Church University’s Ethics Committee. The research followed the British Psychological Society Code of Human Research Ethics.^59^

## Results

### Participants

To minimise the possibility that women might become identifiable, only age groups and services accessed were associated with pseudonyms to preserve anonymity (Table 1). All seven participants were White British, and their average age was 42 years, ranging from 20s to 50s. Length of contact with MH services ranged from 1 to 19 years (average of 8 years). Five women had left their employment due to DVA experiences. Two women worked part-time, and two were unemployed and received financial support through state benefits. Three women lived in refuges, one in temporary accommodation, and three in private accommodation. Five were mothers, and two had had their children taken into care. All had experienced DVA within heterosexual relationships and from a male partner; one participant had also experienced DVA within her family of origin. Abuse duration ranged from 10 to 27 years. All women had separated from their partners at the time of interview; however, many still had contact with the perpetrator through child-contact or legal proceedings.

**Table 1.** Participant information.

Pseudonym	Age group	Services accessed
Louise	40–49	Crisis and home treatment team; DVA refuge.
Caroline	50–59	CMHT; Private psychology; DVA service.
Lauren	20–29	CMHT; Crisis team; Inpatient service; DVA refuge.
Alice	50–59	CMHT; IAPT; Crisis team; DVA service.
Amy	40–49	CMHT; Private psychiatry and psychology; IAPT; DVA service.
Demi	30–39	Crisis team; DVA refuge.
Phoebe	40–49	CMHT; Crisis house; Crisis team; DVA refuge and service.

CMHT: community mental health team; DVA: domestic violence and abuse; IAPT: improving access to psychological therapies.

## Findings

The analysis is presented through three main narratives: ‘missing the connection(s)’, ‘missing windows of opportunity’ and ‘finding a safe haven’. Ten subthemes described women’s accounts and have been presented in italics throughout the text. Phrases in quotation marks are direct quotes from women. Additional quotations are provided in Table 2.

**Table 2.** A table of themes, subthemes and corresponding quotes.

Theme	Subtheme	Quote
Missing the connection(s)	Diagnosed and discharged	‘You try and kill yourself. . . You then get put on to the crisis team, they’re there with you like intensively for a couple of weeks and then it’s like “thanks for coming it’s been emotional, see you later” after the two weeks, nothing’ [Louise].
	Lack of human connection	‘When you’re sectioned like they just come in to medicate you or give you your food. . .there’s no like question like, ’how are you? How are you doing?’. . .nothing. . .their communication skills are just not there. . .at all. They think that because we’re numb from like, all of this. We don’t deserve just normal human interaction, which is really hard’ [Lauren].
	Missing the connection between abuse and distress	‘[I just wanted] someone to unpick it and ask me what is going on? We can see by your records that you’ve got worse over the years, what’s happened? She didn’t use to have a drinking problem. . .What the fuck has happened for her now to be drinking a litre bottle of vodka a day?. . .Why can they not put two and two together with this? They need someone to be able to look at that and think that screams domestic abuse’ [Demi].
		‘I was in a bad relationship with domestic violence, I was using. . . to try and numb the pain. . . it makes you feel unworthy as a person because you’re a substance abuser, that our mental health is totally just put down to substance abuse. . . the mental health comes from the abuse and the substance abuse. It’s just a triangle of shit, you know?’ [Demi].
	Searching for an explanation	‘When I was diagnosed. . .it was a feeling of ‘okay, so I do have something’. So I’m not entirely crazy, because I thought I was going mad. So when I was given the anxiety, depression, and emotionally unstable personality disorder. . ., it was like okay, I do get those feelings and this is why I feel the way I feel and. . .it was kind of refreshing to hear that I did have something, because I just thought that I was just going crazy’ [Phoebe].
Missing windows of opportunity	Invisibility of abuse: Ignored and discounted	‘The MH couldn’t even see it. And if somebody else can’t see it, then why should I deserve to get help?’ [Phoebe]. ‘At no point was the issue was it even broached? It was kind of like all about me. And it was all about my mental health, which I suppose is what they do, it was all focused on me, changing my thought processes, . . .at no point did it address being . . . within abusive relationships. . .I don’t think really, you’re gonna get better. . . It was going to just manifest in a different way’ [Caroline].
	Unhelpful assumptions and labelling	‘People outside of domestic violence and abuse, think that well, why don’t you just leave? Why don’t you just put that drink down? Look listen, if it was easy as that we wouldn’t have been in these relationships for so many years. . .So you know, that is how I feel the mental health team. . . .they look at you as “just put the vodka down and get out of the relationship”. You know, and they think that that’s the answer to everything’ [Demi].
	Inappropriate support	‘They kept kind of promising that something’s going to happen, and nothing did. So apart from every month, you’d get a phone call. . .going through all these questionnaires, exactly the same questionnaires as before, nothing had changed. You were still at the highest level of everything. But they weren’t offering anything. Nothing at all’ [Alice].
	Reaching breaking point	‘I reached out to Crisis team saying that I was mad. . .I was seeing things, I was manic, . . .I was scared. Somebody rang me back. . ., and just said to reach out to A&E in the next 4 to 8 hours. I was like, I can’t get myself to hospital. . .I was too afraid to even leave my house. So I overdosed’ [Lauren]. ‘I just wish that somebody would have noticed the signs. Noticed the bruises. . .the really bad MH. Noticed the weight loss. . . the weight gain. . . the self-harm. . . the self-medication. . .the alcoholism. . . the cannabis use. . . and maybe ask me, “Are you okay?”, “Is everything okay?”’ [Phoebe].
Finding a safe haven	Connection and security	‘When I went privately I didn’t feel like I’d ever lost that support. I could access it again, whenever. . . and it was going to be easy to access. [When] you have to jump through so many hoops, it becomes tiring, when you have been in these relationships, and you’re beaten down, it’s tiring and you don’t want to be going in and having to explain yourself over and over again. . .I think that I feel much safer in the sense that I can now come back and access counselling again or I can call up and talk about things if I if I need to with a service that is going to be able to understand the issues that I’ve got now, or might have if I get into a relationship in the future’ [Caroline].
	Just getting it	‘You need the help with. . .understanding what they’re doing. . . what the perpetrator is doing. Understanding how that makes you think and feel, understanding how to reduce it and all of that. That’s what makes the difference’ [Amy].

MH: mental health.

### Missing the connection(s)

Throughout women’s narratives of navigating and receiving services, there was disconnection between their episodes of care, relationships with MHPs and experiences of DVA and distress, with women often describing searching for an explanation for their distress.

Women faced consistent barriers to accessing and re-accessing support. Many described high thresholds for accessing support resulting in long waits, yet they were quickly discharged, on access. Despite experiencing significant distress, women reported being ‘left’ by services, with no follow-up care or onward referrals, increasing their sense of isolation. Interactions with services were experienced as disconnected, with little consideration of longer-term care planning or integrated support. Instead, women were *diagnosed and discharged* following psychiatric medication prescription. Women then had to fight to re-access support, often in the context of gatekeeping by services. Many felt as though they were ‘going round in circles’ and not being listened to.

‘When I got released from there [hospital], that was it. Had no aftercare. No follow ups. . . . Nothing at all until I then next overdosed and woke up in hospital’ [Lauren].

The brevity of appointments and time-limited nature of support offered women no opportunities to form meaningful, trusting relationships with staff, causing a *lack of human connection*. This was exacerbated by high staff turnover. The focus was often on completing questionnaires or attending online groups rather than face-to-face interactions, exacerbating participants’ isolation and disconnection. The gender of professional also influenced women’s sense of safety and connection. All felt that this reflected the current NHS context where high demand and limited resources result in brief, superficial interactions with professionals.

‘I just. . .didn’t connect with the psychologist. . . when you’re going through domestic violence its quite difficult to open up to a male. . .and he was just asking me questions, like, from a piece of paper and just like ticking a box. . . . So I wasn’t able to disclose everything that I wanted to disclose to him’ [Phoebe].

MHPs consistently showed a lack of understanding and knowledge regarding DVA. There was a lack of curiosity about why women were distressed, and interactions with staff were described as feeling like a ‘tick box exercise’. Women often felt that their difficulties were taken at ‘face value’ without professionals ‘looking deeper’. Professionals therefore often *missed the connection between abuse and distress.*

‘There should have been a breakdown. . .with the crisis team. . . how I got to that stage where I wanted to hang myself and. . .throw myself out of the window. Like, why? What happened in the run up to this point, you know, but they weren’t interested in that. They were just interested in ticking boxes’ [Demi].

MHPs also consistently missed connections between DVA, substance use and MH, with substances often being used as a coping strategy to ‘numb the pain’. Several women described self-medicating with substances due to a lack of service provision but later being denied MH services due to substance use. Some women reflected that MH services appeared to reject any complexity and were more interested in categorising and ‘washing their hands’ of them rather than considering how difficulties might intersect. Demi described her experience as ‘being thrown in a box’ and professionals ‘throwing away the key’.

‘If you’re going through domestic abuse and use drugs. . .you’re instantly looked at as bad. And they don’t really look deeper as to why you’re actually using them. What are you trying to hide? What pain are you trying to heal by yourself but not getting the help?’ [Lauren].

Whilst the experience of DVA led all women to experience significant distress, many did not recognise this connection and were instead *searching for an explanation* for their distress when they initially accessed MH services. For many, this was due to the ambiguity, isolation and confusion created by coercive control tactics: women had a sense of ‘something not being right’ but could not identify what this was, and so considered themselves ‘the problem’. Being called ‘mad’, ‘crazy’, ‘paranoid freak’ and ‘nutcase’ by the perpetrator meant that many women questioned their sanity. Many, therefore, initially welcomed a MH diagnosis as it provided an explanation for how they felt and countered the perpetrator’s narrative that they were going mad.

‘Having the diagnosis obviously doesn’t heal you. . .but I now know, I’m not feeling great. So what’s going wrong? Whereas before, I just thought ‘he’s right, I’m going mad. . . And I didn’t have that real understanding of what’s happening to me’ [Lauren].

Louise described searching to understand ‘why she was the way she was’. She found not receiving a diagnosis invalidating, as MHP’s offered no alternative explanation for her distress.

‘I wasn’t saying, ‘Oh, I’ve got bipolar’ . . . I suppose in a way. . .you want to be labelled. . .but you don’t. But you want a reason to be like, ‘Why am I?’. . .Clearly there is something wrong with me’ [Louise].

### Missing windows of opportunity

Missing the connection(s) had several consequences for women, including being offered interventions that were inconsistent with their needs. Windows of opportunity for meaningful support were, therefore, consistently missed, often increasing women’s distress and their risks of experiencing harm and abuse.

Women described how a context of terror, walking on eggshells, and ‘firefighting’ at home impacted their ability to disclose to others. Women who had not disclosed abuse described the significant risk and vulnerability involved, and how safety and trust were necessary for disclosure to occur. Women often ‘[tested] the water’ with professionals by leaving hints or ‘being vague’ in the hope that professionals would notice, and often wanted professionals to ask them about abuse explicitly.

‘I just felt scared. I felt maybe they wouldn’t believe me. Maybe they thought I was going crazy. . . I was scared in case he found out. . .what he would do to me. It was the stigma around it as well. I felt guilty, I felt ashamed. . . I just wanted somebody to say to me like “what is going on?. . . that’s what I wanted, but that didn’t happen”’ [Phoebe].

For women who did make overt disclosures or explicit hints, or where the abuse was documented within their care records, this were often ‘*ignored and discounted*’ by MHPs. This lack of interest into the context of their distress was experienced as active avoidance and as a lack of care by MHPs. This compounded the stigma and ‘silence’ often associated with experiencing abuse. Amy described how, despite disclosing DVA to MHPs, she was diagnosed with EUPD after her partner made false allegations against her. The psychological abuse perpetrated by her partner created such distress that it acted as confirmation for an EUPD diagnosis. Professionals then viewed Amy’s rejection of this diagnosis as further evidence of a ‘personality disorder’. This was experienced as collusion with the abuse.

‘Because the level of what he was doing was so psychologically, like tormenting. . .it looks like extreme anxiety, it looked like mood instability. . . It wasn’t, it was fear. . . he created the symptoms he wanted them to see’ [Amy].

Focusing on the ‘diagnostic presenting problem’ centred the problem within women, perpetuating the ‘*invisibility of abuse’* and meant the intervention became all about them – rather than the perpetrator and the abuse. For some, this facilitated self-blame narratives and prevented identification of risk.

‘It makes you think that it’s your fault. That actually there’s something wrong with you. . . .that what’s going on at home was fine. . ., and that you’re overreacting. . . or you’re assessing it wrongly. . . especially trying to pull it down to personality disorder. That was the worst’ [Amy].

Alice, was at ongoing risk from a perpetrator, yet was blamed for not controlling intrusive thoughts about being unsafe.

‘He [MHP] undermined me and didn’t take me seriously and was basically saying it was my fault that I was having so many thoughts because I wasn’t doing the right things. . .he made me feel just like my abuser did, that it was my fault’ [Alice].

Missing these windows of opportunity either by not recognising women’s attempts to ‘test the water’, by ignoring women’s previous disclosures, or by not asking about the abuse directly, exacerbated women’s sense of being undeserving of support and perpetuated the stigma and feelings of shame associated with experiencing abuse. For some women, this also resulted in collusion with the abuse and an increase in harm.

Louise had asked MHPs not to give her psychiatric medication to her partner. This was ignored, resulting in him using access to medication as another method of control.

‘They passed it [medication] on to the abuser to give him full control of my tablets. Even though I said I didn’t want that but. . .to them, I’m the nutcase. . . who just keeps trying to kill herself. . . that was totally ignored. . . . it comes across like they don’t give a shit’ [Louise].

Some women therefore strategically managed their interactions with MHPs, keeping the abuse invisible for their own safety, due to not feeling safe within MH services.

‘It used to break me because I thought “oh my god, I just want to tell you. . . it’s on the tip of my tongue”. And, I was just like ‘nah, it’s just not worth it’ [Lauren].

Professionals frequently made *unhelpful assumptions* about DVA, for example, repeating societal narratives about leaving a relationship being a simple problem and failed to acknowledge the systemic barriers to this. MHPs also responded negatively when women advocated for their own needs, and participants reported being aware of the potential consequences if they made complaints or were *labelled* ‘difficult’. Lauren reported being over-medicated in an inpatient ward because she was considered ‘argumentative’. Amy reported being warned that she might be labelled with a ‘narcissistic personality disorder’ diagnosis by the MH team if she continued to challenge the potential EUPD diagnosis. Women, therefore, often felt blamed and judged by professionals, hindering the development of trust and safety.

‘[Services ask me] “Why did you not come to us sooner? If you have been getting abused for seven years?. . .Why is that important? I’m here now. Why are you judging me on not coming? Can you help me now?”’ [Lauren].

Missing windows of opportunity often resulted in *inappropriate support*. All women had been given medication as the initial intervention. Lauren described the irony of being denied MH care due to substance use but then being over-medicated with psychiatric drugs:

‘We can’t help you because you still have drugs in your system’. But. . .you just keep medicating me. How am I ever meant to get off drugs when all I ever know is to have some sort of medication or drug in my system?’ [Lauren].

Lack of appropriate support led to women being offered inappropriate, superficial interventions, which was experienced as MHPs ‘ticking a box’. Louise described being told she needed to self-refer to therapy but feeling unable to do this alone, and ‘to make herself a cup of tea’ by the crisis team when in suicidal crisis.

‘Oh, there is counselling, if you need that it’s a self-referral’. . .the waiting list is extremely long, so if you do have the funds, we would recommend. . .paying privately’. You ain’t going home and telling a violent partner “I need money to go get counselling”. . .It’s basically saying “We’re offering you help with the left hand, however, we’re taking it back with the right hand”’ [Louise].

The power that MHPs held in ‘labelling’ women’s experience of reality resulted in some women feeling trapped in unhelpful and inappropriate services, paralleling their experience of DVA.

‘Once you engage with services. . .for a certain thing. . .it’s a bit of a runaway train. . .I don’t think they go ‘oh no hang on a minute, we’ve got this completely wrong and actually she was being abused’. . . it’s just very hard once you get those things on your medical record, to then change the way people are viewing a situation’ [Caroline].

The lack of responsiveness, silence and repeated missed opportunities from services meant many women *reached breaking point*. Many had attempted to take their own life and/or overdosed on substances. Feeling abandoned by services exacerbated the sense of isolation created by the abuse. Women’s narratives conveyed a sense of loss and ‘what could have been’ if they had received appropriate support when they needed it. Many women lost faith in services and disengaged from the MH system.

‘I feel like if I’d got the help earlier, I wouldn’t have had to go through this nightmare for so long. And in the time that I wasn’t being treated successfully, it just spiralled’ [Alice].

### Finding a safe haven

All participants eventually found a ‘safe haven’ where they felt heard, understood and supported. For most women, this was outside of NHS MH services and within specialist DVA services or private practice and was often later in their journeys of seeking support. Phoebe, however, found meaningful support after moving to a new MH service.

Women described the importance of *connection and security* in their safe haven. The opportunity to build consistent relationships was highly valued and crucial to a sense of safety. For many women, one professional relationship had been pivotal in shaping their journey. Women described the importance of professionals investing in and being alongside them rather than telling them what to do. For all women, being seen and treated with compassion, respect and humanity increased their sense of connection. For many, the context of abuse meant that they often had to access services repeatedly due to fluctuating safety. Caroline reflected that having choice and flexibility over her care was essential to her feelings of security. Women also described how important it was for professionals to hold a sense of hope that things would improve, and this being essential to their ability to keep moving forward.

Phoebe reported that her new MH team was characterised by connection and availability of support: MHPs followed through with what they said, and there was consistency in being ‘listened to’ and ‘treated with kindness’. For some women, peer support (i.e. therapeutic groups, refuge) alleviated the sense of ‘it just being them’. Others highlighted the value of advocacy when they felt completely alone.

‘Being abused, you get told that nobody’s ever going to believe you . . .so you really feel like there’s nothing out there for you. But the staff here and the people I’ve met here. . .you don’t think that they even exist when you’re in that madness you know? So that’s been the biggest support’ [Lauren].

For Phoebe, the consistent relationship with her DVA advocate was crucial to her survival:

‘If I didn’t speak to [DVA advocate], I don’t think I’d be here now. . .it was the way she spoke to me. She was very caring and very worried for my safety that I actually felt heard and listened to the first time in a long time’ [Phoebe].

Caroline’s private clinical psychologist engaged in ‘gentle awareness raising’ at her own pace so that she could reach her own conclusions.

‘There was no pressure on her. . .to kind of tick boxes or. . .try and cram everything in and then end the therapy’ [Caroline].

However, one woman described how the lack of integration with MH services when she was in refuge increased her feelings of isolation and distress.

Women expressed relief at professionals *just getting it* and not having to explain themselves repeatedly. Understanding the dynamics of abuse and providing resources and information put women’s distress into context, named the abuse and alleviated feelings of self-blame and isolation. Women described DVA staff’s intuition, understanding and expertise as allowing them to feel safe in non-blaming, non-judgemental environments and to put their distress in context. Women described the importance of connecting their experiences of abuse with their distress, and the power of recognising that it was not their fault, as a key part of healing.

‘I actually feel like a human. I feel like people listen to me. And here, I’m never judged. . . you feel like you’ve actually got a voice and someone’s listening to you, and someone understands you. You don’t feel alienated at all. . .and I’m starting to realise. . .that it’s not my fault’ [Demi].

As women received more information about DVA, making their experiences more visible, and contextualising their distress, their relationships to diagnosis changed.

‘I’d had such a number done on me. . .I thought I was accessing things for anxiety and flipping, whatever. . .it was because of him. He was never going to. . .go ‘Oh, actually, I think you should go to [DVA service] because I’ve been abusing you mentally and emotionally’. . . You know, it was it was an agenda that was pushed on me’ [Caroline].

‘I just wish that somebody would have said to me. . . you’re going to be okay. You’re feeling like this because you’re being abused. Not because you have emotional personality disorder, not because you’re depressed, not because of your anxiety. . ., because of what you’re going through. . . I didn’t have any of that’ [Phoebe].

## Discussion

This study explored DVA survivors’ narratives of navigating and receiving secondary-care MH services in England. To the best of our knowledge, this is the first narrative study, using the VCRM, to explore DVA survivors’ experiences in secondary-care in the United Kingdom. A narrative approach and in-depth analysis allowed the findings to move beyond exploring the acceptability of routine enquiry and experiences of abuse disclosure to capturing what was important in women’s individual journeys and the challenges and nuances of navigating MH services whilst experiencing DVA. Furthermore, by recruiting through DVA services, the study offers novel findings about the experiences of survivors who may have used alcohol and/or substances to cope and had accessed a variety of UK secondary-care MH services in their journey (e.g. crisis teams, inpatient wards, private practice). This therefore builds upon the findings of previous UK studies that have focused on DVA survivor experiences of CMHTs and did not explicitly include the experiences of those using substances and/or alcohol, which can often be an exclusion criterion for CMHT access.^21,25,26^

The study supports and extends findings that secondary-care MH services in England are not currently conducive to supporting DVA survivors, and the structure and culture of services can replicate the dynamics of abuse, at times exacerbating risk and harm, and too often colluding with perpetrators.^14,20,60^

### Summary of findings

At an organisational level, the structure and culture of MH services left MHPs no time or resources to engage with anything beyond the presenting ‘diagnostic’ problem.^61^ A lack of appropriate support, human connection and meaningful intervention left women feeling ignored and abandoned by MH services, exacerbating the stigma and isolation often associated with experiencing abuse. Consistent with previous UK studies, biomedical dominance also intersected with these constraints to reduce professional curiosity about the context and alternative explanations for distress.^21,25,26^ However, our findings also show that although some women initially welcomed an MH diagnosis as it provided an explanation for how they felt, many described their relationship to psychiatric diagnosis changing over time, and finding it less helpful, as they gained further information about DVA that contextualised their distress. Many women therefore reflected on how diagnosis located the ‘problem’ within them, keeping the perpetrator actions invisible. Women who used alcohol or substances to cope with their distress described sporadic and fragmented support across services due to a lack of consideration of how their difficulties may intersect. Alcohol and substance use was frequently cited as a barrier to MH service access, representing a significant gap in support for DVA survivors who experience long-lasting and high levels of distress and may use alcohol and substances to cope. At the relational level, MHP’s lack of knowledge and understanding about DVA resulted in judgement, blame, and, for some, collusion with the abuse, increasing women’s risk of harm. Women reported wanting to be explicitly asked about DVA, echoing calls for the adoption of routine enquiry within UK MH services.^21,25,26^ An additional finding in the current study was that women also experienced not being asked explicitly about DVA by MHPS as a lack of interest or care, which perpetuated the ‘silence’ around the abuse. Healthcare professionals have reported lacking confidence when enquiring about DVA.^62^ However, participants in the current study reported that even when they did disclose or DVA was noted in their records, they were not taken seriously or listened to in their requests for support, despite making multiple attempts to end their lives. This is in the context of high rates of suicide among individuals who have experienced DVA.^12^

Within the study, women repeatedly cited the importance of caring, consistent relationships in navigating and receiving services, despite system constraints. Relationally, women welcomed feeling heard, being involved in collaborative decision-making, working with staff who understood abuse dynamics, and being able to access appropriate, timely support. This builds on established findings that interventions that take power away from women are not conducive to healing and that ‘RICH’ relationships with staff (i.e. relationships that provide respect, information, connection and hope) can make a significant difference.^5,63,64^ This also supports recommendations from trauma-informed theorists who have argued for the creation of trauma-informed healing environments that are based on safety, respect and dignity, and the promotion of ‘power-with’ rather than ‘power-over’ relationships within MH services.^36,63^

In the current study, the MH system’s unpredictability, uncertainty, lack of responsivity (silence) and enforcement of arbitrary conditions for the provision of care, paralleled women’s experiences of DVA. The power differential and lack of control intrinsic to their abusive relationships was often also replicated in women’s interactions with MHPs. Those who were able to access support within private practice had more positive experiences due to the flexibility, choice, and ability to go at their own pace (rather than time-limited sessions), yet this option is restricted to those with economic means and/or who are not in situations where using private services could intensify DVA. The current findings support existing evidence that failure by MH services to respond appropriately to DVA, either by discounting the violence or by providing inappropriate support, can place women at risk of ongoing and escalating violence and increased distress.^65^ These issues have been reported in various settings.^18–21,66^ The wider context to such settings in the United Kingdom is one of austerity, lack of resources, high staff turnover and burnout amongst the workforce.^19^

### Implications

The current findings have clinical implications that echo the wider global and UK-based literature.^14,19–21,25,26^ Despite the widespread implementation of policies, guidelines, and training within the last decade in MH settings in the United Kingdom, concerns regarding harm and re-traumatisation of DVA survivors continue to persist. This suggests that a wider cultural shift involving systemic change within MH services is required, including adopting trauma-informed approaches, and considering alternative conceptualisations of distress beyond diagnosis.^35,67,68^

Given that one in four women experience DVA in their lifetime, and that DVA both causes and exacerbates mental distress, MHPs will have regular contact with individuals who have experienced or are currently experiencing DVA.^2^ Therefore, the findings support calls for continued DVA training and engagement with MHPs in order to increase understanding and confidence in responding appropriately to DVA disclosures.^14^ The findings also highlight MHPs power in shaping women’s journeys, as well as the importance of human connection in reducing feelings of shame and isolation. It is important to emphasise within training that existing interpersonal and clinical skills mean MHPs are well-placed to support DVA survivors, build relationships, and provide thoughtful and compassionate support.

Like participants in previous studies, women reported wanting to be asked explicitly about abuse so that windows of opportunity for appropriate support were not missed.^21,25,26^ A novel finding in the current study was that survivors experienced a lack of enquiry about DVA from MHPs as active avoidance and a lack of care. This extends previous findings to suggest that not only is routine enquiry acceptable and encouraged in MH services but also when MHPs do not ask about DVA this can be experienced as unacceptable and even harmful.^21,25,26^ MH services are arguably well-placed to explore this within initial assessment and compassionate enquiry is particularly required when supporting those who have attempted to end their lives. The Lara-VP resource provides useful guidance for UK MH services on how to implement this.^69^

A key factor in the lack of curiosity about the context of women’s distress was the dominance of the biomedical model. The study supported findings that the language and dominance of the biomedical model had profound implications for how women initially understood and made sense of their experiences. Often the only explanation available within MH services was a medicalised one. For those who had explicitly disclosed DVA to MH services, many found that their diagnosis was often used to discredit their experiences of abuse, and to challenge their credibility. This is consistent with research showing that survivors are often stigmatised within MH services, and that women in particular, have frequently been dismissed due to their MH diagnoses and labelled as ‘hysterical’ or ‘mad’ when disclosing abuse.^4,19,23,25,39,70^ This strongly indicates that alternative conceptualisations of distress beyond diagnosis are required, and MHPs would benefit from reflecting on power, privilege, and assumptions related to DVA and substance use, and how these factors may impact the care they provide.^19,23,25^

The principles of trauma-informed care – which focus on the importance of relationships, and rather than a diagnostic focus ask, ‘what happened to you’ and ‘what do you need?’ – may be a helpful guide to inform MH practice. The UK government has defined trauma-informed practice (TIP) as recognising that trauma is prevalent and can affect individuals, groups and communities; recognising the signs and widespread impacts of trauma; and seeking to prevent re-traumatisation, which is the re-experiencing of thoughts, feelings and sensations related to traumatic experiences.^71^ TIP is based on several core principles including: safety, trust, choice, collaboration, empowerment, and cultural consideration. All too often, participants in the current study had experienced a lack of these within MH services. Despite MH services often claiming to be trauma-informed, a core aspect of TIP that is arguably neglected is the need for systemic change, including structural changes to policies and procedures, and staff training and support. Without meaningful structural change, MH services risk only offering a change in language, whilst continuing to focus and locate trauma at an ‘individual level’, perpetuating adverse experiences for DVA survivors.^72^

An essential part of TIP may therefore be ensuring that MH services are specifically DVA-informed so that MHPs can identify and respond appropriately to survivors’ distress in context. Adoption of an advocacy approach when considering DVA within secondary-care MH services may also reduce staff pressure to ‘intervene and fix’, and instead encourage MHPs to sit alongside, listen to, and consider survivors’ expertise in exploring valuable support.^62,73^

Participants themselves had important recommendations for mandatory training, awareness of support, support pathways, integrated support, relationships, and being listened to and believed (Table 3). In particular, women wanted to see greater integration across DVA, drug and alcohol and MH services to support increased collaboration, sharing of knowledge and the development of clear referral pathways across services.^6,31^ Integrating knowledge and increasing resources across services and adopting trauma-informed approaches may help to shift the discourse from ‘individual trauma’ into the social and political realm, which could have significant implications for policy and practice, reducing medicalised responses that have consistently been shown to be unhelpful for DVA survivors. Integration across specialist DVA and MH services is already being implemented and has shown success throughout the United Kingdom, through specialist healthcare independent domestic violence advocates. Similar models have also been used to integrate NHS and third-sector services in the United Kingdom within the homelessness sector.^31,74,75^

**Table 3.** Participant recommendations for MH services.

Participant recommendations	Description
Mandatory training	• Training on domestic abuse could be integrated into professional training programmes, or MH services could receive specialist training from DVA services, particularly on coercive control and non-physical types of abuse. • MHPs to be more knowledgeable and understand the signs and dynamics of coercive control. • Increased MHP understanding about the intersections between DVA, MH, alcohol and substance use. • Increased MHP understanding about repeated interpersonal trauma and how this can impact MH.
Development of a pathway	• It was suggested that a development of a pathway for DVA survivors in MH services would be helpful, so that women can receive timely support, given how quickly their circumstances may change and windows of opportunity can be missed. • More specialist support within MH services for DVA was recommended.
Integrated support	• Women asked for more integrated support between MH teams and DVA services (e.g. MH support being available when women enter refuge). • The development of links between MH and DVA services was recommended to improve knowledge and clinical practice.
Consistency of relationships	• Where possible, consistency with the same worker should be prioritised within MH services to provide the opportunity to build relationships and trust over time. • Women highlighted the importance of having a plan in place (e.g. for when MHP’s are on leave) and knowing an MH worker they could contact in their absence.
Awareness of support	• Many women described not knowing what support was available to them. They therefore suggested that it may be helpful for information about coercive control and domestic abuse to be displayed within individual clinic rooms, waiting rooms, and MHPs should have resources that can be shared with women, if it is safe to do so.
Listening and being taken seriously	• Most importantly, women recommended that it would be helpful for MHPs to reflect on their own assumptions, to not judge others, listen, and take their concerns seriously. • Women also emphasised the importance of their own expertise being respected within MH consultations.

DVA: domestic violence and abuse; MH: mental health.

Despite this, there is a gap in provision for individuals who experience DVA and who also experience long-lasting and high levels of distress and/or use substances and/or alcohol.^65^ This can result in fragmented and inappropriate care for those trying to access support. DVA services in the United Kingdom often receive unstable and short-term local authority funding and although an increase in resources and funding would be important to allow DVA services to continue to offer valuable and specialist support, DVA and gender-based violence is a public health issue that MH services will continue to encounter, given the prevalence of DVA across society and the ongoing impacts on people’s MH.^8,29^ Furthermore, as seen in this study, many women are not in a position to disclose DVA when they initially come into contact with MH services and therefore may not be referred to specialist DVA services at initial contact. It is arguable that without more integrated support, services will not be able to meet the reality of diverse needs for many survivors who experience long-lasting and high levels of distress. The involvement of and collaboration with DVA survivors and professionals in the co-construction of policy and MH service development will be essential.

### Strengths and limitations

A key study strength was the qualitative design and in-depth analysis, including four readings of each individual narrative, which facilitated immersion into each woman’s journey through services. Comprehensive theme identification was enabled by comparing and contrasting narratives across several stages and iterations of thematic analysis. As this was a narrative project focusing on individuals’ experiences, the number of included participants was informed by previous studies that had used a similar design (e.g. VCRM) and that had focused on DVA.^51,60^ This is consistent with the study of Mauthner and Doucet who argued that a ‘saturation point’ is reached where authors have enough data to contribute to the work.^50^ The study adopted a trauma-informed approach, involving collaboration with DVA survivors and staff. This shaped the project design, interview questions and the development of a standard operating procedure, increasing the safety of participation. Furthermore, member checking helped to ensure that the findings were consistent with participant experiences. A reflexive approach was also taken, and the lead author’s positioning as a feminist researcher and adoption of a trauma-informed approach meant that a curious, non-expert stance was adopted, encouraging open engagement with the data and listening and learning from women’s narratives rather than reporting what the lead author ‘already knew’. However, it is inevitable that the lead author and supervisor’s standpoints contributed towards how the data were understood and the emerging analysis was constructed.

Women were recruited from specialist DVA agencies and therefore may have compared their experiences of MH services with their experiences of specialist support, potentially shaping their narratives and the conclusions drawn. Their narratives may therefore differ from survivors who have accessed secondary-care MH services but not specialist DVA services, and from individuals who possibly had fewer positive experiences within specialist DVA services.

All participants identified as cis-gender, White British and heterosexual, and therefore, experiences of Black and minoritised, and LGBTQ+ individuals may differ, especially given the structural inequalities that are known to impact experiences of accessing and receiving both DVA and MH support.^23,76,77^ There are currently significant demands and reductions in funding for UK specialist DVA provision. Initial recruitment was supported by a service that the lead author had an existing relationship with to support the safety and to develop the trauma-informed approach of the project. This perhaps limited the representativeness of the sample. Further services (including ‘by and for’ DVA services) were contacted to support the representation of a range of experiences; however, due to current constraints, these services were unable to participate. The current sample therefore may reflect wider structural barriers and systemic health inequalities that are known to impact Black and minoritised women’s and LGBTQ+ people’s experiences of accessing both DVA and MH support.^78^

### Recommendations for future research

Whilst the current research is unique, it also echoes over 25 years of literature that DVA survivors’ can experience healthcare services as unhelpful and re-traumatising, and as paralleling their experiences of abuse.^61^ Despite these repeated findings, there has been limited practical or cultural change within MH service provision.^19,79^ Some researchers are therefore turning to methods such as participatory action research to directly impact DVA survivors’ experience and close the research–implementation gap.^26,80^ Future research with Black and minoritised survivors, including people who identify as LGBTQ+, is urgently needed. Women in this study also reported eventually reaching a ‘safe haven’ where they could begin to heal. Further research into understanding the journeys and pathways to ‘safe haven’ for those experiencing DVA and long-lasting and high levels of distress is recommended. Furthermore, this study focused on DVA survivors’ experiences of secondary-care MH services. It would be important for future research to explore survivors’ specific experiences of both primary and tertiary-care MH services to establish how findings may be similar to or different from those presented in the current study.

## Conclusions

The study’s findings provide further evidence for previously expressed concerns about the ability of secondary-care MH services in England, as currently constituted, to provide safe and appropriate support to DVA survivors, despite the widespread implementation of policies, guidelines and training within the last decade in MH settings in the United Kingdom.^15–17,21,25,26^ The structure and culture of MH services were found to parallel and collude with abuse dynamics, exacerbate risk and harm, and re-traumatise DVA survivors. This suggests that a wider cultural shift involving meaningful systemic change within MH services is required, including adopting trauma-informed approaches, more integrated support and considering alternative conceptualisations of distress beyond diagnosis. Given that one in four women in the United Kingdom will experience DVA in their lifetime, this is a highly prevalent public health issue that MH services must be better equipped to support.

Despite system constraints, the importance of caring and consistent relationships with professionals in which women were listened to and treated with compassion and humanity were consistently cited as making a difference. This means that individual practitioners can have transformative impacts, even within trauma-uninformed systems.

## Supplemental Material

sj-pdf-1-whe-10.1177_17455057251336142 – Supplemental material for ‘Where is the help for me here?’ Female domestic violence survivors’ narratives of navigating and receiving secondary-care mental health services in the United Kingdom Supplemental material, sj-pdf-1-whe-10.1177_17455057251336142 for ‘Where is the help for me here?’ Female domestic violence survivors’ narratives of navigating and receiving secondary-care mental health services in the United Kingdom by Megan Webb, Anne Cooke and Angela Sweeney in Women’s Health
